# Sino-European Transcontinental Basic and Clinical High-Tech Acupuncture Studies—Part 2: Acute Stimulation Effects on Heart Rate and Its Variability in Patients with Insomnia

**DOI:** 10.1155/2012/916085

**Published:** 2012-03-04

**Authors:** Gerhard Litscher, Guangyu Cheng, Weiping Cheng, Lu Wang, Qianqian Niu, Xiao Feng, Ingrid Gaischek, Haixue Kuang

**Affiliations:** ^1^Research Unit of Biomedical Engineering in Anesthesia and Intensive Care Medicine and Stronach Research Unit for Complementary and Integrative Laser Medicine, TCM Research Center Graz, Medical University of Graz, 8036 Graz, Austria; ^2^Heilongjiang University of Chinese Medicine, Harbin 150040, China

## Abstract

This second part of a series of Sino-European high-tech acupuncture studies describes the first clinical transcontinental teleacupuncture measurements in patients with insomnia. Heart rate (HR) and heart rate variability (HRV) measurements in 28 patients (mean age ± SD: 41.9 ± 14.6 years) were performed under standardized conditions in Harbin, China, and the data analysis was performed in Graz, Austria. Similar to the first part of the series, the electrocardiograms (ECGs) were recorded by an HRV Medilog AR12 system during acupuncture of the Shenmen point (HT7) on the left hand. HR decreased significantly (*P* < 0.001) during and after acupuncture stimulation of the HT7 acupuncture point. Total HRV increased significantly (*P* < 0.05) immediately after acupuncture stimulation, but there was no long-lasting effect. The values of the low-frequency (LF) and high-frequency (HF) band increased significantly after the stimulation compared to baseline values; however, the LF/HF ratio showed no significant changes. Together with the results of previous studies, the present results can serve as a solid basis for further development of acupressure or acupuncture stimulation equipment for complementary use in treating insomnia.

## 1. Introduction

Insomnia is a common condition in which the patient has trouble falling or staying asleep. It can range from mild to severe, depending on how often it occurs and for how long. It has become a global health problem. In the scientific database PubMed (http://www.ncbi.nlm.nih.gov/pubmed/), there are more than 2,300 reviews on this topic. One of these articles, published recently in the Journal of Clinical Sleep Medicine [1], indicates the necessity for further research on the relationship between the effects of acupuncture on insomnia and autonomic regulation, which might guide better selective use of this treatment modality for insomnia [1].

This second part in a series of transcontinental high-tech acupuncture studies deals with the acute effects of manual needle acupuncture on heart rate (HR) and heart rate variability (HRV) in patients with insomnia. The aim of this study was to test the hypothesis that patients with insomnia will demonstrate decreased HR and increased HRV acute effects during and after acupuncture treatment as measured by electrocardiographic monitoring and spectral analysis techniques. Similar to previous studies on patients with depression [2] and poststroke patients [3] in Harbin, patients with burnout [4], and animal experimental investigations in Beijing [5], a transcontinental teleacupuncture design was used. This means that the data were recorded in patients in China and analyzed in Austria [4, 6].

## 2. Materials and Methods

### 2.1. Patients

In total, 28 patients (5 male, 23 female) with a mean age of 41.9 ± 14.6 (SD) years (range: 22–82) were investigated in this transcontinental study. They all presented themselves at the hospital due to insomnia. The Athens Insomnia Scale (AIS) was used for classification of the disease [7]. The scores ranged from 6–21, resulting in a mean value of 12.4 ± 3.6 (SD). The subjects had no obvious history of heart disease or cerebrovascular disease, respiratory, or neurological problems. The patients were fully informed about the nature of the investigation, and they all provided their informed consent. The methodological procedure and registration of the noninvasive parameters were approved by the local ethics committee and in accordance with the Declaration of Helsinki of the World Medical Association.

### 2.2. Electrocardiographic Monitoring

Bioelectrical cardiographic (ECG) activity was recorded using an HRV Medilog AR12 (Huntleigh Healthcare, Cardiff, UK, and Leupamed GmbH, Graz, Austria) equipment. The data were analyzed using new “Fire of Life” software (Huntleigh Healthcare) [4, 8]. The sampling rate of the recorder is 4096 Hz, allowing R-waves to be detected extremely accurately, and a monitoring period of more than 24 hours is possible. All raw data are stored digitally on a 32 MB compact Flash memory card. After removing the card from the portable system, the data are read by an appropriate card reader connected to a standard computer and sent to the research unit in Graz. The dimensions of the HRV recorder are 70 × 100 × 22 millimeters, and the weight is approximately 95 grams with batteries [8]. ECG registration was performed in Harbin with three adhesive electrodes (Skintact Premier F-55; Leonhard Lang GmbH, Innsbruck, Austria) applied to the chest.

HR and HRV, which is the percentage change in sequential chamber complexes called RR-intervals, can be calculated from the ECG. HRV can be quantified in the time and frequency domains using ECG power spectra [8–11]. These parameters are recommended by the Task Force of the European Society of Cardiology and the North American Society of Pacing and Electrophysiology [11]. The mean HR, total HRV, LF (low-frequency), and HF (high-frequency) bands, and the LF/HF ratio of the HRV were evaluated [11].

### 2.3. Acupuncture Stimulation and Procedure

**Figure 1:**
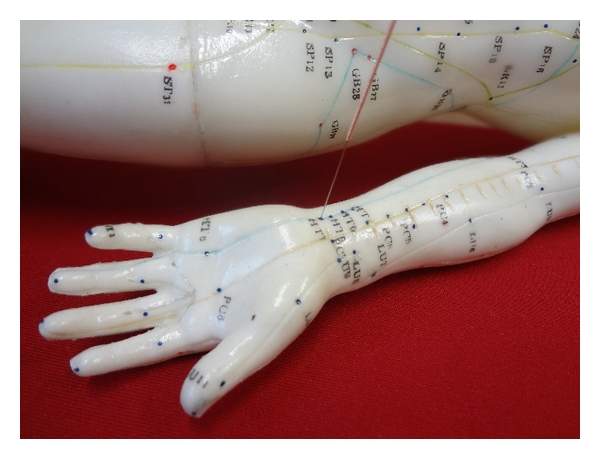
Shenmen (HT7) acupuncture point stimulated with a metal needle.

The “Shenmen” (HT7) acupuncture point on the left arm was selected for stimulation. Shenmen is located on the wrist, at the ulnar end of the crease of the wrist, in the depression of the radial side of the tendon of the ulnar flexor muscle of the wrist (see Figure 1). This acupuncture point is indicated mainly in cases reporting cardiac pain, restlessness, and insomnia [12].

**Figure 2:**
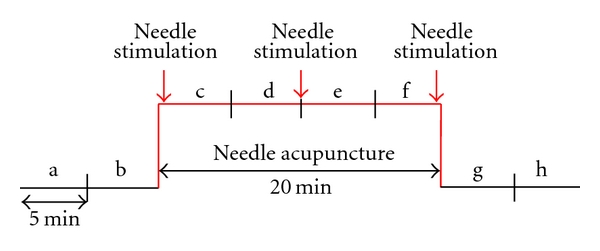
Experimental protocol for manual needle acupuncture at the Shenmen acupuncture point.

For manual acupuncture stimulation, sterile single-use needles (length: 30 mm, diameter: 0.3 mm; Huan Qiu, Suzhou, China) were inserted perpendicularly to the skin to a depth of approximately 15 mm at the acupoint. The needles were stimulated clockwise and counterclockwise for 15 seconds each, with two rotations per second, resulting in 30 rotations per stimulation. The stimulation was performed immediately after inserting the needle, 10 minutes later, and before removing the needle (see Figure 2). The measurement profile and measurement times (a–h) are shown schematically in Figure 2. Eight measurement periods were compared: two before stimulation (a, b), four during acupuncture (c–f), and two after acupuncture (g, h).

### 2.4. Statistical Analysis

The data were analyzed using one-way repeated measures analysis of variance (ANOVA) or Friedman repeated measures ANOVA on ranks (SigmaPlot 12.0, Systat Software Inc., Chicago, USA). Post-hoc analysis was performed using the Tukey and Holm-Sidak tests. The level of significance was defined as *P* < 0.05.

## 3. Results

**Figure 3:**
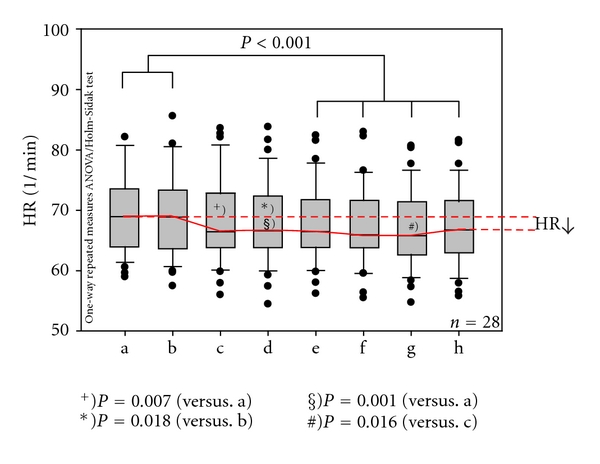
Box plots displaying the mean heart rate (HR) of the 28 patients. Note the highly significant decrease beginning in phase (e). The ends of the boxes define the 25th and 75th percentiles with a line at the median and error bars defining the 10th and 90th percentiles. The different measurement phases (a–h; compare with Figure 2) are indicated.

**Figure 4:**
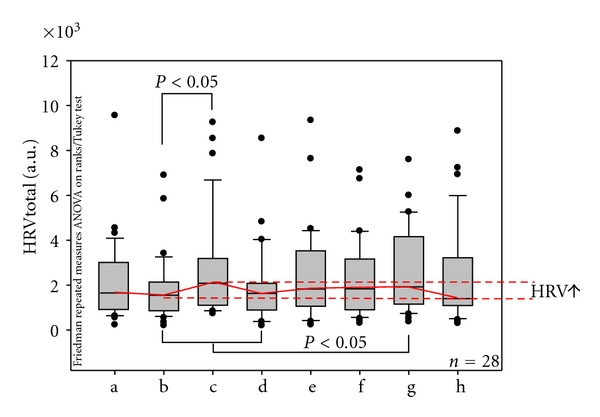
Changes in total heart rate variability (HRVtotal) before, during, and after needle stimulation at the Shenmen acupoint. For further explanation, compare with Figure 3.

Mean HR and total heart rate variability (HRVtotal) are shown in Figures 3 and 4. In these figures, the results from 28 patients for measurement phases a–h (before, during, and after stimulation of the Shenmen acupoint) are documented. There was a highly significant (*P* < 0.001) decrease in HR after the second needle stimulation (phase e) compared to the two control intervals (a, b) before stimulation. This effect remained manifest throughout the rest of the stimulation (phase f) and during the control intervals after stimulation (g, h).

In contrast to HR, total HRV increased significantly (*P* < 0.05) in two intervals immediately influenced by needle stimulation (c, g). However, this was not a long-lasting effect; at the end of the measurement period (approximately 10 minutes after the last stimulation), values had returned to baseline.

**Figure 5:**
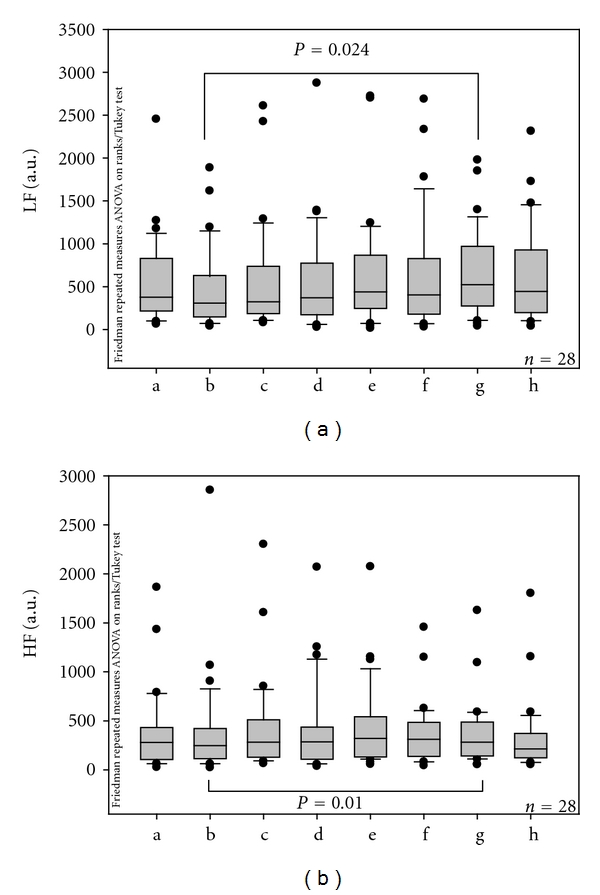
Values of the low-frequency (LF) and high-frequency (HF) bands. Note the significant increase in both bands. For further explanation, compare with Figure 3.

Figure 5 shows the values of the LF and HF bands within the different measurement phases. The values describing the LF and HF bands increased significantly in the interval immediately following the last needle stimulation compared to the interval before the first needle stimulation.

**Figure 6:**
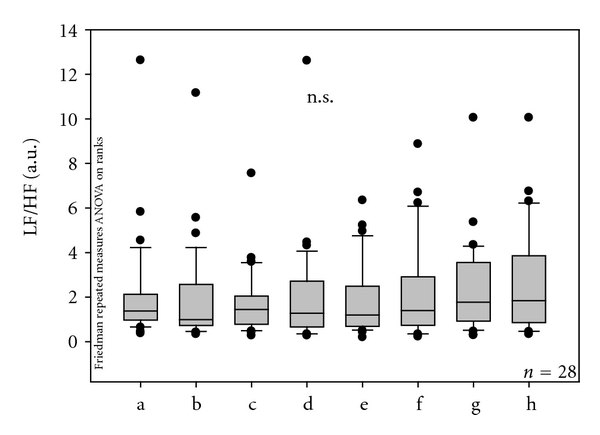
Ratio of the LF (low-frequency) and HF (high-frequency) band of HRV in the 28 patients before, during, and after needle stimulation. No significant alterations were found. For further explanation, see Figure 2.

Furthermore, continuous HR-HRV monitoring showed no significant changes in the LF/HF ratio (Figure 6).

## 4. Discussion

In recent years, computer analysis of heart rate and its variability has allowed for the identification of specific brain-modulated autonomic influences, which reflects the effects of individual mechanisms involved in cardiovascular regulation. New systems and tools for evaluating the features of cardiovascular control have been developed [8, 9]. The application of these tools in acupuncture research should lead to a deeper understanding of the regulation mechanisms and also to the quantitative assessment of the effects of acupuncture stimulation. Evidence has also been provided that HRV may have prognostic value in different diseases involving autonomic dysfunction [9, 11].

Beat-to-beat variations of human heartbeat intervals (HRV) have also been investigated using spectral analysis during acupuncture [10]. Mean HR, total power of HRV, power in the LF and HF band, and a normalized power ratio of the LF and HF bands are parameters that somehow reflect sympathetic and/or vagal modulating influences on heart rhythms [10, 11]. In this study, the acute influence of manual needle acupuncture stimulation (Shenmen acupuncture point) on these parameters was investigated under standardized conditions in patients with insomnia. As already mentioned, acupuncture has been shown to modulate the activities of the sympathetic and the parasympathetic nervous systems, which are essential for cardiovascular function. This fact has led to its clinical use in the management of various diseases [9–11].

In 1995, Lin [13] stated in the journal *Psychiatry and Clinical Neurosciences* that acupuncture is a simple and useful treatment for insomnia. He reported a success rate of approximately 90% [13], stating that one of the most effective points is the Shenmen (HT7) body acupoint. To review trials on the efficacy of auricular acupuncture treatment for insomnia, 878 publications were included in a meta-analysis by Chen et al. in 2007 [14]. In all studies (100%), the most commonly used auricular acupoint was the Shenmen acupoint. The authors stated very critically that most trials were of low quality, and therefore, clinical trials with better design quality and a longer duration of treatment are necessary. In our study, we investigated the effects of the Shenmen (HT7) hand acupuncture point. It would be very interesting to perform a separate study investigating the effects of the corresponding or not corresponding Shenmen ear acupuncture point. This was also one of the reasons why we performed a one-point acupuncture study for the first time.

There are several studies concerning acupuncture and insomnia that randomly divided the patients into test groups and control groups [15–18]. Altogether, the rate of effectiveness was higher in the experimental groups. Therefore, all authors concluded that the symptoms induced by insomnia were significantly improved, which is why we did not choose a control point in our study. It has also been demonstrated in a paper published by Ruan in 2009 [19] that the sleep quality of insomnia patients can be significantly improved by acupuncture. Yeung et al. stated in *Sleep* [20] that there is an advantage of electroacupuncture over placebo acupuncture in the short-term treatment of primary insomnia. However, due to the small advantage and some shortcomings of their study, the five authors are not sure if there is a real benefit of electroacupuncture in the treatment of insomnia.

To clarify and elucidate the underlying mechanism involved in the use of acupuncture to treat insomnia, different parameters have to be investigated. Recent studies investigated serotonin and malondialdehyde levels, which are markers for oxidative stress in depressed patients with insomnia [21]. The authors found that the serotonin pathway is involved in the pathophysiological mechanism and that this could be influenced by acupuncture [21]. Other parameters investigated in this context were the blood flow velocity in the middle cerebral artery, basilar artery, and vertebral artery. The cerebral blood flow velocity was increased in this study in the control group, with a more obvious increase in the observation group [22]. In addition to the biosignal parameters, different scores can be used. The findings of recent Chinese investigations published in 2010 showed that acupuncture can improve insomnia patients' clinical symptoms [23, 24].

In an interesting prospective, randomized, placebo-controlled double blind cross-over study, polygraphic monitoring was performed during night sleep in six healthy volunteers [25]. Acupressure at the Shenmen (HT7) hand acupoint was used. After one night of adaptation, two PEBA cones (Polyether Block Amides; Isocones) were fixed bilaterally at HT7 or on the back of the hand (placebo application). Sleep efficiency increased in patients treated with verum acupressure as demonstrated by a decrease in wakefulness and increased their total sleep time as demonstrated by an increase in non-REM (rapid eye movement) sleep [25]. To the best of our knowledge, this is the only study using stimulation of a single acupoint (Shenmen, needled bilaterally) in the context of insomnia. The significance of our data indicates that maybe differences exist between those patients who respond to acupuncture insomnia therapy and those that do not. Further research into the use of electrocardiogram and other physiological parameters to stratify response to acupuncture therapeutic interventions is warranted. Together with the results of our present study, this report can serve as a solid basis for the further development of acupressure or acupuncture stimulation equipment for additional use in treating insomnia.

## 5. Conclusions

The following conclusions can be drawn from the results of the present transcontinental teleacupuncture study in patients with insomnia.

- Heart rate decreased significantly during and after acupuncture stimulation of the Shenmen acupuncture point on the left hand.
- Total HRV increased significantly immediately after acupuncture stimulation, but there was no long-lasting effect.
- The values of the LF and HF band increased significantly after the stimulation compared to baseline values; however, the LF/HF ratio showed insignificant changes.
